# Barriers and enablers to secondary use of multi-regional linked administrative health data for three distinct user groups: academic researchers, health-system knowledge users, and private sector researchers

**DOI:** 10.23889/ijpds.v9i5.2894

**Published:** 2024-09-18

**Authors:** Raquel Duchen, Donna Curtis Maillet, Ted McDonald, Jodi Gatley, Keltie Gale, Minnie Ho, Michael Schull, Trish Caetano, Anne Hayes

**Affiliations:** 1 HDRN Canada; 2 ICES; 3 New Brunswick Institute for Research, Data and Training, UNB; 4 Keltie Gale Consulting; 5 Canada's Drug and Health Technology Agency (CADTH)

## Objectives

We analyzed barriers and enablers to accessing pan-Canadian health sector data and data analytic services for three distinct user groups: 1) academic researchers, 2) health-system knowledge users (KUs), and 3) private sector researchers. Specifically, we a) conducted a legislative and policy scan regarding use of health administrative data for research, quality improvement, and health system management and b) systematically consulted with data centre directors and staff, privacy and legal experts, knowledge users (KUs), private sector entities, and university and government officials.

## Results

KUs and private sector organizations face unique barriers because structures and regulations related to data access and use are set at multiple university and government institutions and are primarily designed for academic research. Additionally, data repositories often have specific policy/legislative barriers to working with KUs and/or private sector researchers. However, all groups face common issues including variations in regional regulations and policies making harmonized and streamlined processes challenging; lengthy data access and ethics review processes; and regional variations in data availability. Enablers included diverse expertise and initiatives across Canada such as: initiatives to streamline ethics reviews for multi-regional research, expand data holdings, increase standardization and harmonization, and implement systems for federated analyses.

## Conclusions

Access to health system data and analytics in Canada varies by user group and across regions. Although each user group faces unique challenges, they also all have many barriers and enablers in common.

## Implications

Improving health system data access for one use group has the potential to benefit the others.

